# The Effects of Physically Embodied Multiple Conversation Robots on the Elderly

**DOI:** 10.3389/frobt.2021.633045

**Published:** 2021-03-22

**Authors:** Toshiaki Nishio, Yuichiro Yoshikawa, Kazuki Sakai, Takamasa Iio, Mariko Chiba, Taichi Asami, Yoshinori Isoda, Hiroshi Ishiguro

**Affiliations:** ^1^Department of Systems Innovation, Graduate School of Engineering Science, Osaka University, Osaka, Japan; ^2^Faculty of Engineering, Information and Systems, University of Tsukuba, Tsukuba, Japan; ^3^NTT DOCOMO, Inc., Tokyo, Japan; ^4^NTT Media Intelligence Laboratories, NTT Corporation, Kanagawa, Japan

**Keywords:** human-robot interaction, elderly people, conversational robot, multiple robot, embodiment of robot, physical robot, virtual robot

## Abstract

In recent years, communication robots aiming to offer mental support to the elderly have attracted increasing attention. Dialogue systems consisting of two robots could provide the elderly with opportunities to hold longer conversations in care homes. In this study, we conducted an experiment to compare two types of scenario-based dialogue systems with different types of bodies—physical and virtual robots—to investigate the effects of embodying such dialogue systems. Forty elderly people aged from 65 to 84 interacted with either an embodied desktop-sized humanoid robot or computer graphic agent displayed on a monitor. The elderly participants were divided into groups depending on the success of the interactions. The results revealed that (i) in the group where the robots responded more successfully with the expected conversation flow, the elderly are more engaged in the conversation with the physical robots than the virtual robots, and (ii) the elderly in the group in which robots responded successfully are more engaged in the conversation with the physical robots than those in the group in which the robots responded with ambiguous responses owing to unexpected utterances from the elderly. These results suggest that having a physical body is advantageous in promoting high engagement, and the potential advantage appears depending on whether the system can handle the conversation flow. These findings provide new insight into the development of dialogue systems assisting elderly in maintaining a better mental health.

## Introduction

Recently, the aging population has increased worldwide (United Nations, 2019). One of the problems in an aging society is the lack of social contact for the elderly. It has been reported that the degree of social contact affects the mortality of the elderly (Goldman et al., 1995; Berkman and Leonard Syme, 2017). Furthermore, it was reported that lonely people were more likely to experience a decline in activities with regards to daily tasks and that loneliness was associated with an increased risk of passing early (Perissinotto et al., 2012), implying the importance of mental support for the elderly. Our ultimate goal is to develop a robot offering mental support for the elderly by acting as their conversation partner. To achieve this, conversation systems with multiple robots have been developed (Iio et al., 2017; Arimoto et al., 2018). As it seems to be suitable for our purpose, we adopted an extended version of the multiple conversation system (Iio et al., 2020b).

There are many existing studies on the effect of robot embodiment in various settings: some showed the advantage of having a physical body (Heerink et al., 2010; Deng et al., 2019) whereas others showed the opposite (Looije et al., 2010). It is worth noting that the conversations carried out in these studies were task-oriented, with the purpose of accomplishing a particular task. On the contrary, to deepen the relationship with people, a different type of conversation, such as casually asking people's experiences, is expected to be accomplished (Clark et al., 2019). However, it is not clear which type of robot the elderly prefer in such a type of conversation. Therefore, this paper investigates how the behavior of the elderly and their feelings change depending on whether the interlocutor robots have physical bodies or not in the experience-asking conversation.

Although it was limited to a text-based conversation with younger participants, Kiesler et al. reported that their engagement in conversation with a physical robot was more enhanced than a virtual one (Kiesler et al., 2008). Unlike their study, we consider fully verbal conversations for evaluation, which seems to be a more user-friendly way for the elderly than the text-based one. We conjecture that the positive engagement of the elderly in the fully verbal conversation will also be reproduced in terms of both behavioral aspects. Thus, we test the following hypothesis:

(H1) The elderly are more engaged in the experience-asking conversation with physical robots than with the virtual robots.

By contrast, Kiesler et al. also reported that people disclose themselves to virtual robots more than physical robots (Kiesler et al., 2008). Consistently, although it was not examined in the experience-asking conversation, Looije et al. showed the negative effect of having a physical body on the conversation, kindness, and friendliness of the robot (Looije et al., 2010). Therefore, we predict that the total impression about the relationship with the robots, such as closeness to them, is less positively evaluated for the one with physical bodies than the one without them. Namely, we test the following hypothesis:

(H2) The elderly evaluate virtual robots as closer interlocutors than physical ones in the experience-asking conversation.

To verify these hypotheses, we implemented two conversation systems: one with multiple physical robots and one with multiple virtual robots. We conducted an experiment in which participants aged from 65 to 84 compared these systems. Even though the multiple robot conversation system (Iio et al., 2020b) is expected to work to avoid conversational breakdown, it is still difficult to perfectly control the quality of conversation among participants because of the unexpected recognition of their replies. In the analysis, therefore, we consider how successfully the robot detects expected words in human replies. This paper reports the results of the experiment and discusses their implications.

## Related Works

The notion of embodiment has attracted the attention of researchers in artificial intelligence (Ziemke, 2001), which covers multiple concepts not only regarding the properties stemming from the physical body of the agent but also the coupled structure between its body and environment that can be considered even when it does not exist in the physical world. In the context of human-robot interaction, one of the advantages of having a physical body is that it enables a robot to interact with the environment, including humans, which provides services by physically moving and making contact with objects in the world, including humans, such as guiding visitors (Nourbakhsh et al., 2003) and assisting the elderly people (Pollack et al., 2002). In addition, it is also reported that the physical body influences the non-physical aspects of the interaction. For example, a robot with a physical body was perceived as more credible and informative than the one with a virtual body because of its physical presence (Kidd and Breazeal, 2004). Li asserted that physically present robots were more persuasive and perceived more positively than those digitally displayed on a monitor screen with or without its photo-realistic appearance (Li, 2015). On the other hand, such a virtual representation can be considered as one constituting another possibility as an influential body for a conversational robot. Holz et al. argued that a virtual agent has some merits (Holz et al., 2009). It can act even in a physically impossible way, such as mutating their form (Martin et al., 2005). Meanwhile, they can also exhibit a high degree of anthropomorphism by using highly expressive representations, which can be easily adjusted and personalized for users without expensive cost compared to the agent with physical bodies (Johnson and Rickel, 1997; Kopp et al., 2005).

Regarding the role of the physical body of a social robot, Deng et al. reviewed past robotics research and summarized the bodies that were adopted in various tasks and how they were evaluated (Deng et al., 2019). From the review, the conclusions regarding the type of robot preferred differs depending on the type of task. For example, a physical robot received more attention than a virtual robot and the interaction with it was more enjoyable for people in some situations, such as playing chess (Leite et al., 2008; Pereira et al., 2008), solving a puzzle (Wainer et al., 2007), and storytelling (Costa et al., 2018). In contrast, it was not preferred in other situations. For example, in lecturing scenario, people memorized less contents from the lecture given by a physical robot than those by a virtual robot (Li et al., 2016). The social presence of the physical robot was more positively evaluated than the virtual robot where the physical touch was allowed in the interaction, while it was more negatively evaluated where the physical touch was restricted (Lee et al., 2006). In a conversational interaction, in which a robot persuaded people, they perceived a virtual robot as more competent than a physical robot (Hoffmann and Krämer, 2013). Kiesler et al. reported that university students engaged more in conversations with physical robots than with virtual robots, while they did not disclose socially negative behavior to physical robots as much as to virtual robots (Kiesler et al., 2008). These studies indicate that the physical and virtual robots have their own benefits and it is important to choose the type of robot depending on the purpose.

There are many studies reporting the positive effect of physical embodiment in task-oriented human-robot interaction on the elderly, while others have reported the opposite. The physical robot provided more positive influences than the virtual robot in coaching physical exercise (Fasola and Mataric, 2013) as well as in guiding music therapy (Tapus et al., 2009). In conveying the information, Heerink et al. reported that the physical robot more effectively informed the elderly of the alarm and weather forecast than the virtual one (Heerink et al., 2010). On the contrary, it was reported that the virtual one outperformed the physical one in advising the elderly on their health (Looije et al., 2010). It is worth noting that the conversations carried out in these studies were task-oriented conversations. In the field of study for the elderly, the effect of the physical body of the robot in a more social conversation, such as casually asking people's experiences, has not been clarified. Therefore, in the current study, we investigated the difference between the interaction with physical robots and that with virtual robots in the experience-asking conversation.

## System

### Multiple Robots

To be a conversation partner for the elderly, robots need to be programmed with the ability to talk autonomously and naturally as humans with them. For this, certain challenges such as accurate speech recognition and answer generation in conversation with the elderly need to be addressed (Young and Mihailidis, 2010). A classical but effective and less costly approach is making a robot reply with ambiguous reactions (e.g., “I see.” and “I understand”) independent of the conversation; this has been used more or less in previous conversation systems (Weizenbaum, 1966; Wallace, 2009). However, if a robot keeps on repeating such replies, users may assume that the robot does not understand the context of the conversation and that the robot ignores them. Arimoto et al. proposed a method to reduce such a negative impression by switching the speaker and the bystander role among multiple robots (Arimoto et al., 2018). The effectiveness of this method was confirmed even in a field experiment (Iio et al., 2017). Furthermore, Iio et al. extended the conversation system with multiple robots to perform robust conversations with the elderly by including a function of proxy response to maintain the conversation even when an elderly person does not respond (Iio et al., 2020b). Multiple robots were allowed to ask open questions to the elderly; this provided a robot-initiated but more freewheeling conversation.

We developed a question-answer-response dialogue model (Iio et al., 2020b) extended with an active listening function, which encouraged the elderly to speak in certain ways. In parallel with this study, we conducted a field experiment in a facility for the elderly and a laboratory experiment and investigated whether the elderly's speech increases by adding the listening function. In this study, we focus on the potential effects of the physical bodies of a multi-robot. This section describes the system implemented in the experiment.

### Question-Answer-Response Dialogue Model

**Figure 1:**
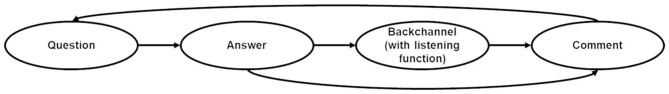
State transition diagram.

The question-answer-response dialogue model is a model developed to continue to interact with a person even with low speech recognition accuracy. It has four states as illustrated in Figure 1: (i) Question state where the system asks a question to the person (e.g., “Have you ever been abroad?”), (ii) Answer state where the person answers it (e.g., “I don't remember.”), (iii) Backchannel state where the system shows a brief acknowledgment to the human answer (e.g., “I see”), and (iv) Comment state where the system expresses its opinions and impressions to the human answer (e.g., “I would like to go aboard.”). The system begins with the question state and moves to Answer, Backchannel, and finally Comment state in this order. After that, it starts again with Question state and follows the same sequence. By repeating this, the system continues the conversation with the person. In the exceptional cases when no answer is detected in the Answer state, the system skips Backchannel and transits to Comment state. In the Backchannel and Comment state, the system utterances are generated by choosing one from several patterns depending on the recognized answer in the Answer state.

For example, in the above question about travel, assume that the system has supposed that the person replies with either answer formats such as “yes, I have _____” and “no, I haven't.” Therefore, if the person answers “yes, I have been to Hawaii,” the system finds the phrase “yes, I have” in the format and produces a corresponding backchannel such as “sounds nice” in the Backchannel state and a corresponding comment such as “you have a wonderful experience” in the Comment state. If the person answers “no, I have not. But, I would like to go abroad,” the system finds the phrase “no, I haven't” and utters “oh, you have not? However, there are many people who haven't been abroad, right?” When there is no matching phrase, the system randomly selects one from the prepared general sentences in the Backchannel and Comment state such as “I see” and “I haven't been abroad, so I want to go there,” respectively.

In the system, the two robots spoke alternately. When one robot in the Question state queried a person, the other robot showed a backchannel in the Backchannel state. Subsequently, the robot that asked in the Question state produced a comment in the Comment state. In the next Question state, the two robots alternated roles with each other. In this way, we intended to equalize the numbers of utterances of the two robots.

### Listening Function

In the question-answer-response dialogue model (Iio et al., 2020b), the system identifies the end of speech of the person when no utterance has been detected for a certain period in the Answer state and then moves to the Backchannel state. It sometimes causes an error, i.e., the system detects the end of speech even when the person intends to continue speaking but inserts a relatively long pause during his or her speech. Therefore, we have developed a listening function to try promoting a person's speech when it detects silence so that it avoids terminating his or her speech before he or she finishes. Specifically, the system produces not only a backchannel but also an utterance to promote the person to speak more (e.g., “Please tell me more about it”). It then waits for the person to speak again for 5 s. If he or she utters again, it produces a backchannel again and waits for the person to speak again for 3 s. When it does not detect any utterances within the waiting time, it recognizes that the person has finished his/her speech and shifts to the Comment state. In the Comment state, it selects comments depending on the person's utterance detected in the Answer state and then returns to the Question state. Note that for the purpose of controlling the experiment, the listening function was activated only for predetermined questions.

### Agents

**Figure 2:**
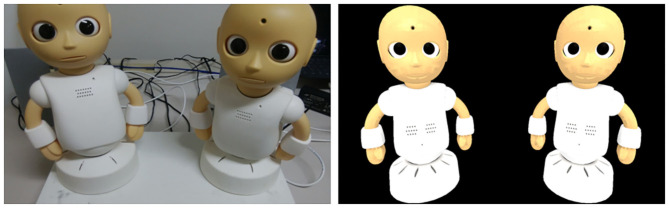
The appearance of CommU (Left) and the virtual CommU (Right).

In this study, we implemented two types of conversation systems: one with physical robots and the other with virtual robots. For the one with physical robots, two desktop size humanoid robots “CommU” developed by Vstone and Osaka University were adopted (Figure 2 Left). CommU has 14 degrees of freedom (DOF): three for the neck, three for eyes, one for eyelid, one for mouth, two for each arm, and two for the waist. However, in this experiment, only eight DOFs, namely three for the neck, three for the eyes, one for the mouth, and one for the waist were utilized with the aim of practical application at a low cost in the future. When CommU receives an action command, it produces a sequence of postures that are defined for each command. In this experiment, four action commands were prepared, each of which made it look like tilting its head, nodding, looking at a person, and looking at another CommU. Since the positions of the person and two robots were fixed, the actions for looking were implemented to produce predefined postures of the neck, eyes, and waist so that it looked at either the face of the person or another robot. The commands to open and close the mouth were alternately sent to the speaking robot at a constant tempo during its utterance.

For the virtual robot, two computer graphics characters “virtual CommU” were adopted (Figure 2 Right). The 3D model of the virtual CommU was created by accurately scanning CommU to resemble its appearance. Virtual CommUs were drawn on the web browser by using Three.js, i.e., a JavaScript library for creating 3D content, and WebGL, which is a JavaScript allocation programming interface for rendering interactive 3D graphics on the web browser. They were displayed on the monitor to be of the same size as CommU, while a black background was drawn behind them. They can produce an animation of the same actions as those prepared for CommU. Note that the looking action at the person by virtual CommU was implemented by making it look at the focal point of the scene camera to capture the 3D content so that the person felt being looked at by the virtual robot.

**Figure 3:**
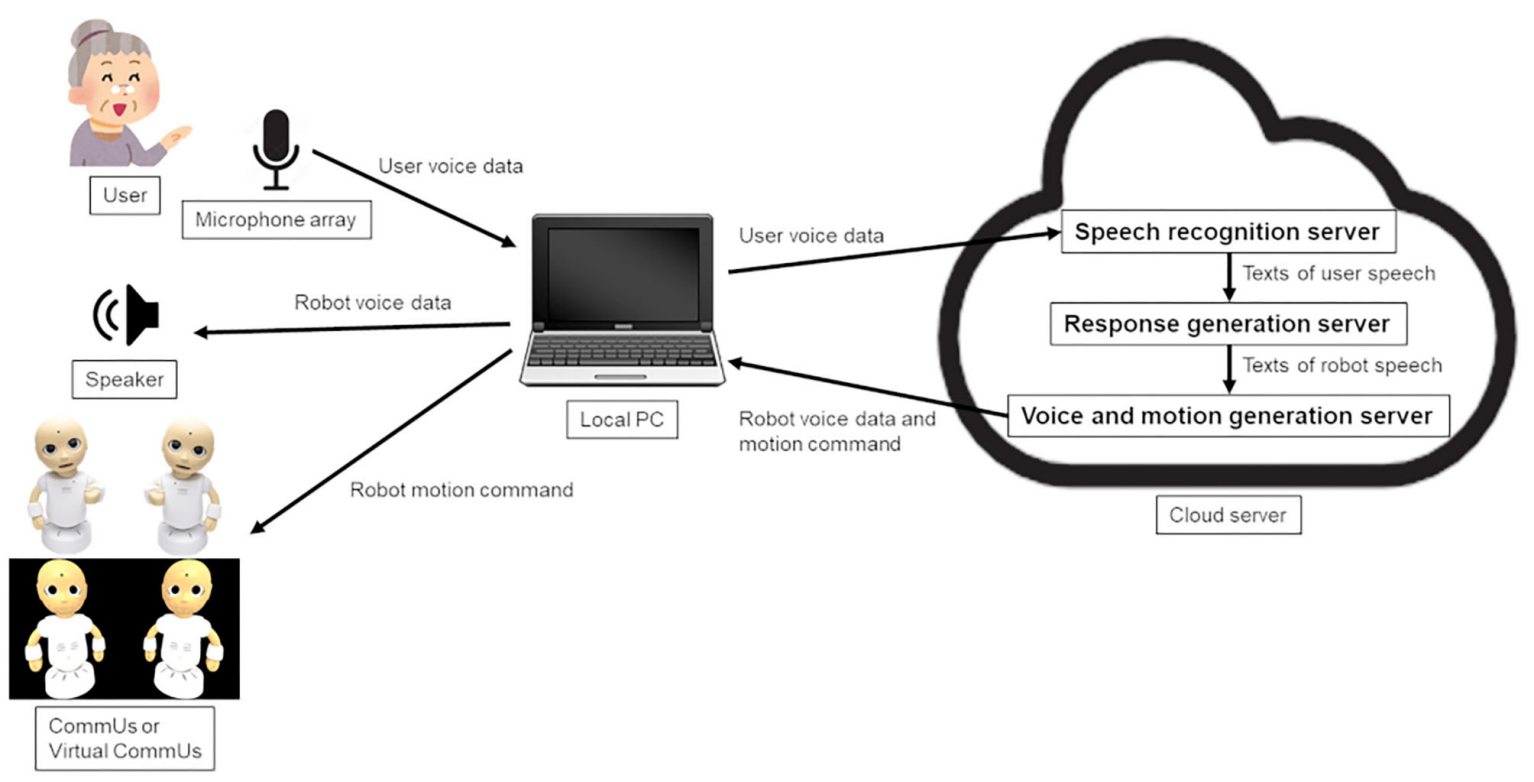
System architecture diagram.

Figure 3 shows the system architecture diagram. Sounds captured by a microphone array were sent to the server program that recognized the speech and sent back the recognized text. Another program synthesized childlike voices and sent them to a terminal computer to play them with a stereo speaker. Although the appearances of the two robots were the same, each robot was made to have different characters so that the person could discriminate between them. Namely, one was given the name of a boy (Taro) and a voice like that of a boy while the other was given the name of a girl (Hanako) and a voice like that of a girl.

## Method

In this study, we carried out an experiment comparing two conditions: physical and virtual. The experiment involved a between-subject design. In the physical condition, an elderly participant talked with a conversation system that operated two physical robots, namely CommUs. In the virtual condition, an elderly participant interacted with the system that operated two virtual 3D characters that resembled CommUs, namely virtual CommUs. The participants were asked to answer the questionnaire after talking with either pair of robots. The experiment was approved by the ethics committee of Osaka University.

### Participants

Forty elderly persons (20 men and 20 women) aged between 65 and 84 years were recruited by a temporary employment agency to participate in the experiment. We included participants who were able to come by themselves to take part in the experiment. Subjectively, the experimenter found no difficulty in communicating with them. We were assured that they had no hearing problems and did not feel excessive fear when confined in a room for the experiment. They were randomly assigned to the physical or virtual condition. Eleven men and nine women were assigned to the physical system, while nine men and 11 women were assigned to the virtual system.

### Apparatus

**Figure 4:**
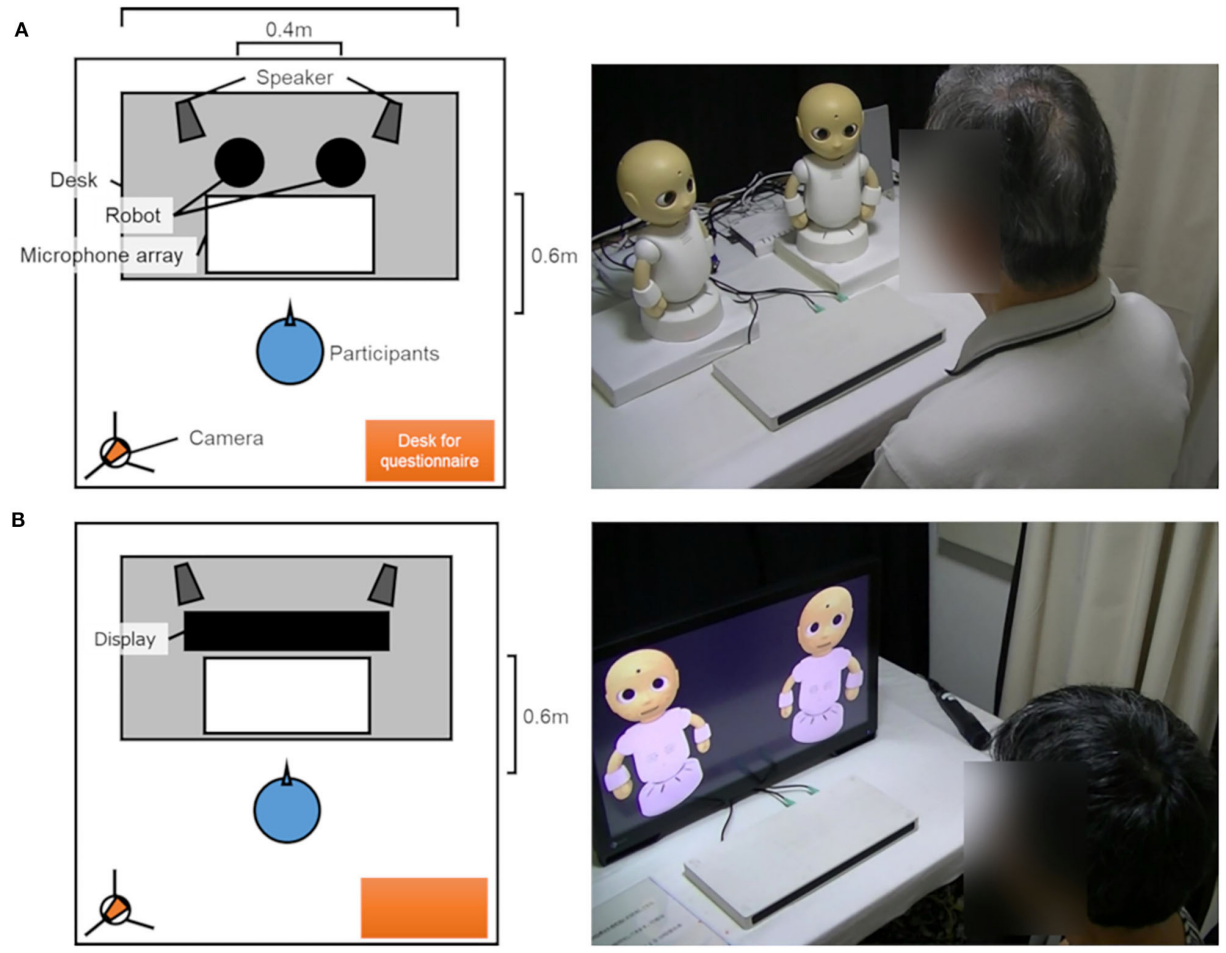
Bird's eye view (left) and scenes (right) of a conversation between a participant and the robots of each condition (**A**: the physical condition, **B**: the virtual condition).

The participant faced either CommUs or virtual CommUs (Figure 4) in an experiment room. The physical robots or the virtual robots, a microphone array, and a stereo speaker were placed on a desk ~1.2 m long. Virtual CommUs were displayed on a 27-inch monitor. A white stage with a height of 30 mm was used to place the CommUs on it so that their height was equal to those of virtual CommUs. The distance between the two robots was about 0.4 m, and the stereo speakers were placed behind each of them. The participant sat 0.6 m away from the robots or the display. A camera was installed on the left back of the participant for an experimenter to monitor the experiment room. A small table was installed at the back and right side of the participant for him/her to answer the questionnaire.

We prepared two conversation scenarios for the experiment. The first set, consisting of five questions, was employed to get the user accustomed to the conversation with physical or virtual robots. At first, the robots introduced themselves and requested the participant to answer questions. Then, they asked questions based on the proposed model described in section Related Works. The topic of the questions was about weather and seasons such as “Is the weather good today?” and “Where do you want to go if you go to a cool place in summer?” For only a limited number of questions, specifically, three out of five questions, the system was allowed to activate the listening function to reduce the burden of answering on the participant. After completing five questions, they said that the training session was over and asked him/ her to wait for a while until the next conversation would start. Note that they terminated the conversation after 5 min even if they did not finish asking all questions.

The second set was used as the experimental stimuli and consisted of 20 questions, each of which belonged to either type of topic: relatively light and serious. The former consisted of 14 questions about childhood memory as well as experience and preference for travel. The latter consisted of six questions about health condition, feelings in daily lives, and expectations or anxiety for the future. Table 1 shows the questions and the order in which they were presented. The robots first asked the participant to answer questions as in the training session and then started asking questions. As with the first scenario, the system was allowed to activate the listening function for only half of the questions on light topics, namely seven out of the 14 questions. On the other hand, the system was allowed to activate it at all questions of serious topics because it was considered unnatural for the robots to not to show interest when the participant responded to such questions. The questions marked with one or more asterisks in Table 1 correspond to the listening function. After finishing them, they thanked the participants for answering their questions. Note that they terminated the conversation after 15 min even if they did not finish asking all questions. The conversation length was determined by a pilot experiment so that we could expect that each participant was allowed enough time to answer the questions for the collection of a sufficient amount of data.

**Table 1:** Questions used as the experimental stimuli.

**Topics**	**#**	**Questions**
Light topic (Childhood memory)	1	*When you were a child, were there many parks?*
	2**	*When you were a child, did you like running?*
	3**	*When you were a child, what kind of toys did you play with?*
	4	*When you were a child, which did you like better, playing outside or at home?*
	5**	*When you were a child, did you like school?*
	6**	*What do you remember most about your elementary school days?*
	7	*When you were in elementary school, did you like studying?*
Serious topic	8**	*Do you want to share the wisdom you've learned in your daily life?*
	9**	*Have you ever had a good time recently?*
	10**	*Do you think about how to live from now on?*
Light topic (Travel)	11	*Have you ever been on a trip and seen a World Heritage site?*
	12	*Which is better for you to travel, foreign country or Japan?*
	13*	*Where is the most interesting place you have ever been?*
	14	*Have you ever eaten soft ice cream while traveling?*
	15*	*When you go on a trip, which do you like better for dinner at a ryokan, Japanese food or Western food?*
	16	*If you go on a trip, don't you think hot spring is the best?*
	17*	*Do you like traveling?*
Serious topic	18*	*Is there anything new you want to start?*
	19*	*Where would you like to go most?*
	20*	*Do you feel sick or worried about your physical condition?*

*The system activated the listening function in the questions marked with one or more asterisks. The questions marked with double asterisks were used for the analysis*.

For each question in both scenarios, some expected user replies were listed. In addition, a backchannel and comment utterances were prepared for each expected word, which were produced when the system detected the user utterance containing it. Meanwhile, another ambiguous comment was prepared for each question, which was used when it did not detect any expected words. Note that nine utterances were prepared to be commonly used as backchannel utterances when the system did not detect any expected words.

Some utterances in the scenarios included special symbols representing the names of the participants. The symbols were replaced with the name of the current participant before the experiment so that the robots could reproduce it.

### Procedure

First, the participant received an explanation about the procedure of the experiment from an experimenter in a waiting room. The participant then moved to the experimental room and sat down in front of the robots. After the experimenter confirmed it through the camera installed in the room, the participant made the system start the first conversation for practice. Then, the system terminated the conversation either when 5 min had passed or all five questions were asked. After that, the experimenter asked the participant to check if he/she found any problem while interacting with the system that needs to be fixed, such as adjusting the volume of the sound. The experimenter then made the system start the next conversation, which was the experimental stimulus. The system lasted the conversation until either when 15 min had passed or when all 20 questions were asked. Finally, the experimenter asked the participant to answer a questionnaire to report their subjective evaluations of the robots.

### Measurement

To evaluate the engagement of the participants to verify hypothesis H1, we measured the average amount of utterance of the participant. We calculated this value by subtracting the total length of silent periods and one of the robots' talking from a duration, which is from when a question had started to when the next question starts. The period of silence was defined as the period when the sound volume was less than the predefined threshold of 0.5 s or longer. Although in the previous study, the amount of time that the participant spent with the robot or agent was measured to evaluate the engagement (Kiesler et al., 2008), we focused on the average amount of utterance of the participant because we had limited time to restrain the participants in the experiment. In this study, we assumed that the participants engaged in a conversation when they used a large amount of utterances.

To evaluate the perceived closeness to verify the hypothesis H2, we used the inclusion of another in the self (IOS) scale (Aron et al., 1992), which has been widely used in previous human–robot interaction research (Mutlu et al., 2006; Cramer et al., 2009; Vázquez et al., 2017). In this scale, the subject is asked to choose a figure to best represent the relationship between him or her and the target agent, i.e., the robots in our experiments, from seven options, each of which consists of two circles and the amount of their overlap represents the degree of closeness. A score was assigned to each figure, namely the figure representing the furthest relationship was one, while the score representing the closest relationship was seven.

### Analysis

To verify H1, we compared the amount of utterances by the participants observed in the conversation with physical or virtual robots. Note that the number of questions the robots could ask in the conversation varied among the participants. To normalize the data, we focused on the participants' answers in the first 10 questions that consisted of both types of relatively light topics and serious ones. Therefore, we excluded the data from participants who answered <10 questions. Among these 10 questions, we focused on answers to seven questions asked with the listening function (the questions marked with double asterisks in Table 1) because it was not easy for the participants to sufficiently interact for questions without the listening function, regardless of the embodiment of robots. To verify H2, we compared the IOS scores for physical and virtual robots.

The engagement and impression of the elderly toward the robot may change depending on how successful the robot is in responding to a speech (Pripfl et al., 2016). The robots generated different responses depending on the participant's answer to their questions. They could produce utterances explicitly about their question if they found words in the participant's answer, which was matched with the expected list. Otherwise, they produced ambiguous ones. As candidate words were carefully prepared to decrease the false positive ratio by not expecting to have a high true positive ratio, it was assumed that the former type of utterance would likely sound more contextually successful. It is considered that the frequency of such successful utterances had a significant impact on the user's impression about the interlocutor. To consider the successfulness of the robots' utterances in the analysis, we divided the participants into two groups, depending on the success of the groups, based on the average number of participants' utterances involving the matched words, which were supposed to induce a successful response from the robot.

In summary, the independent variables of the experiment were the type of robot (two levels: physical or virtual) and the successfulness of the robots' utterances (two levels: more or less). We carried out a two-factor analysis of variance (ANOVA) for the average amount of utterances of the participants and IOS scores.

## Results

The number of participants who replied to over 10 questions were 26 (15 men and 11 women). The average number of successful answers of all participants out of the 10 answers to be focused was 3.54. The participants were divided into the more successful group and the less successful group depending on whether they got more or less successful answers than the average. Table 2 shows the average number of successful answers in each group. The number of participants in the more successful group were 16 (eight each for the physical and virtual conditions), while the number of participants in the less successful group were 10 (five each for the physical and virtual conditions).

**Table 2:** Average number (and standard deviation) of answers that the agents responded successfully.

	**Successfulness**	
	**Less**	**More**
Physical	2.20 (0.84)	4.38 (0.52)
Virtual	1.80 (0.84)	4.63 (0.74)

**Figure 5:**
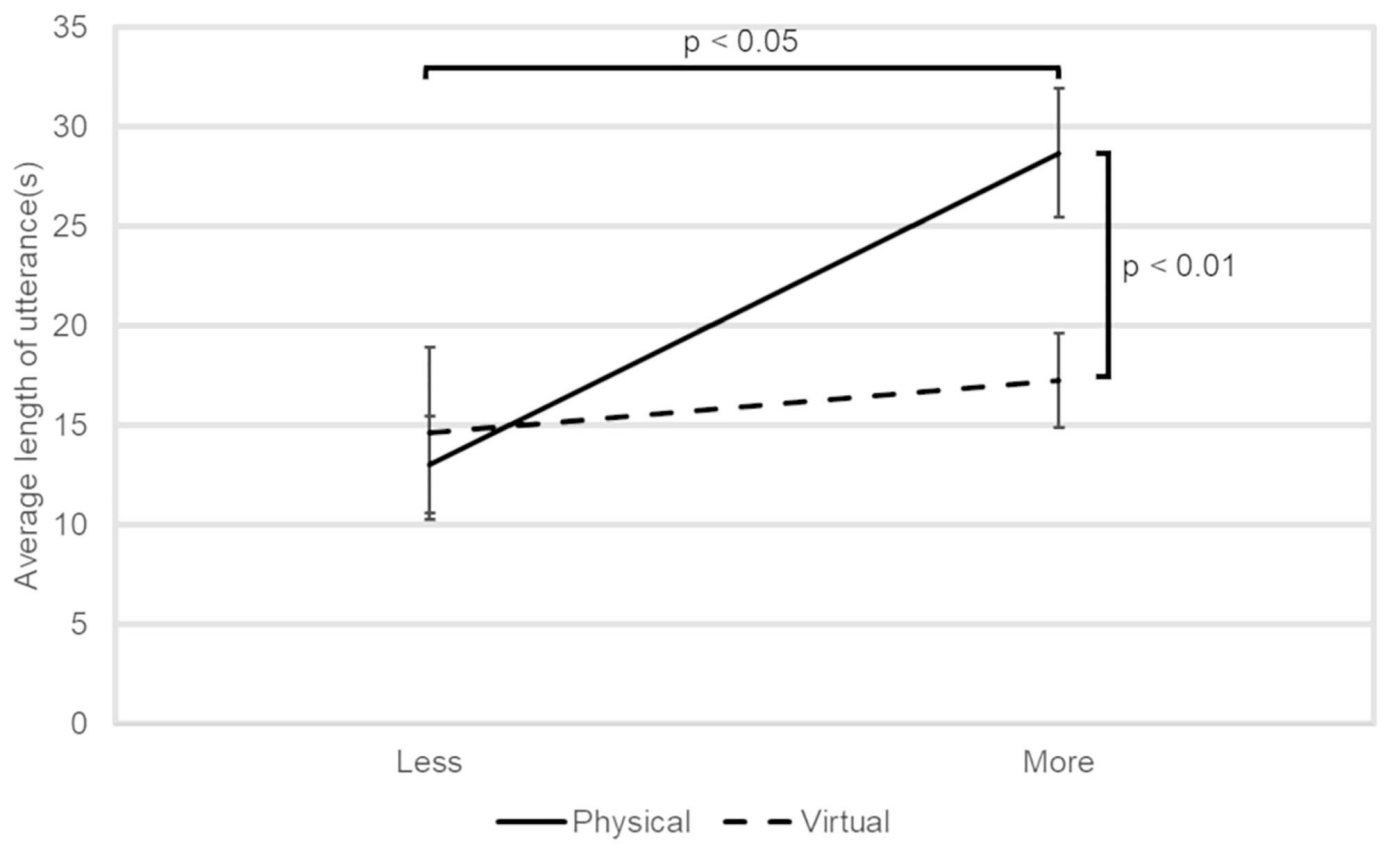
The average amount of utterance of the participants per question.

Figure 5 shows the average amount of utterances of the participant per question. The solid and broken lines represent the physical and virtual conditions, respectively. Error bars denote the standard errors. Two-factor ANOVA showed that the interaction between the type of the robots and the success did not reach at the significant level (*p* < 0.05) but the tendency level [*F*(1, 22) = 3.46, *p* = 0.076, $\eta_{\text{p}}^{2}$ = 0.14]. Analysis of the simple main effect of each factor revealed that the average amount of utterances was longer in the physical condition than in the virtual condition [*F*(1, 22) = 10.018, *p* = 0.0045, $\eta_{\text{p}}^{2}$ = 0.31] for the more successful group. In addition, in the physical condition, the average amount of utterances was longer in the more successful group than in the less successful group [*F*(1, 22) = 6.94, *p* = 0.015, $\eta_{\text{p}}^{2}$ = 0.24]. Note that, the value of the mean squared error was 75.45.

**Figure 6:**
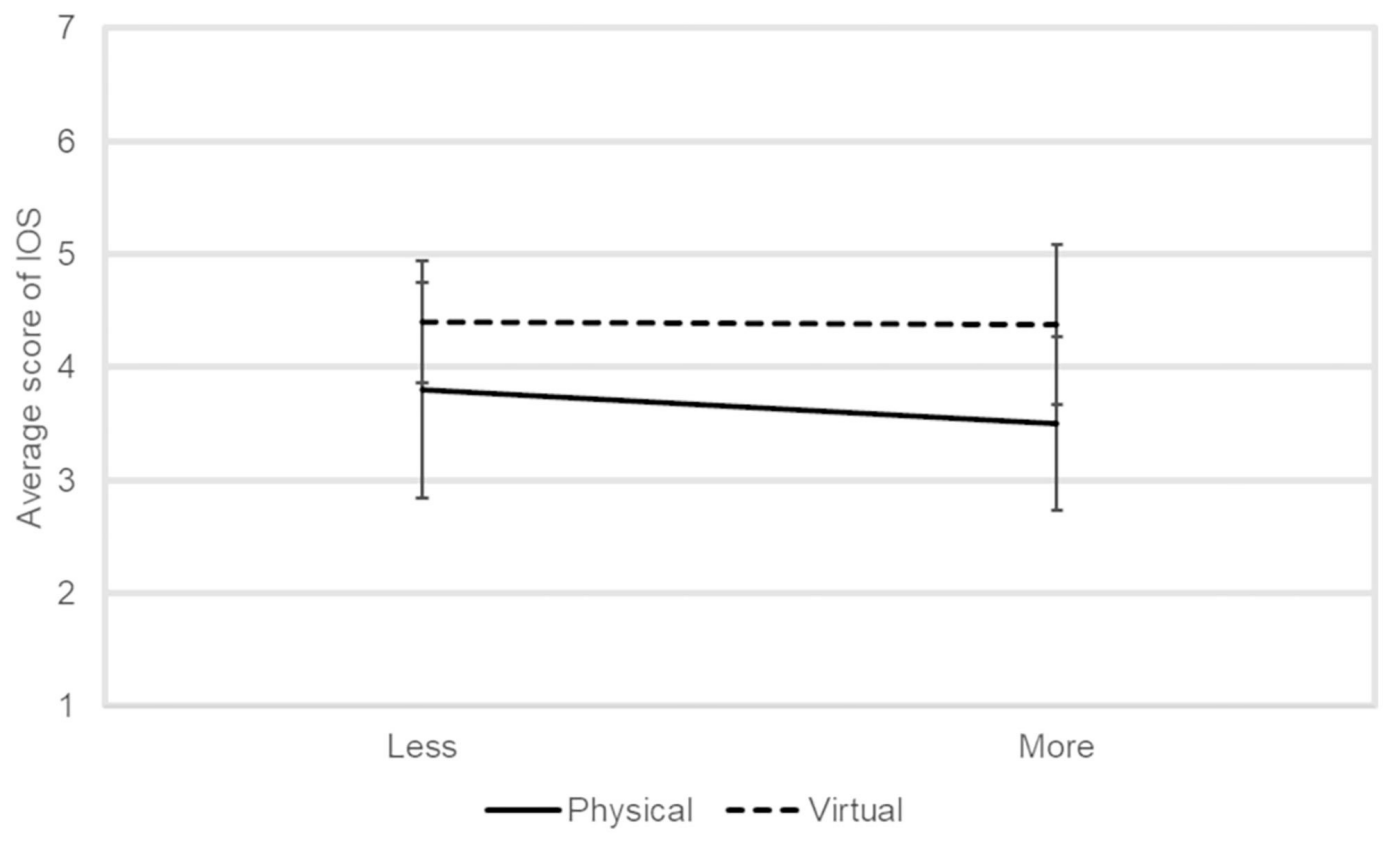
The average IOS score of the participants in each condition.

Figure 6 shows the average IOS score of the participants in each condition. Two-factor ANOVA did not reveal any significant main effects or interaction.

## Discussion and Limitations

Regarding H1, in terms of engagement, it was shown that the elderly engaged in the conversation with the physical robots more than the virtual ones in the groups with more successful responses from the robots, as we expected. In a previous study with university students, it was reported that they were more engaged in conversations with physical robots than with virtual robots (Kiesler et al., 2008), which is in accordance with the current results. Assuming that social abilities of a robot is more effective in enhancing the acceptance of a physical robot than a virtual one (Heerink et al., 2009), participants in the physical robot condition may attribute social abilities to their successful responses more than in the virtual one. It is worth noting that the current result also implies that the benefit of having a physical body becomes more prominent as technologies for successful responses further develop.

H2 could not be confirmed by the subjective measurement. Figure 6 shows that neither physical nor virtual robots are rated extremely high or low. This is not consistent with previous studies showing that virtual robots can be felt as closer to physical robots (Kiesler et al., 2008; Looije et al., 2010). Unlike these studies, the current dialogue system inevitably involved the social interaction between two agents, which demonstrated social abilities and might enhance users' acceptance toward the physical robot (Heerink et al., 2009). This specific effect might conceal the predicted low evaluation of closeness to physical robots.

The present study had some limitations. The virtual robot is limited in its movement and facial expression. Regardless that, one of the advantages of a virtual robot is the capability of arbitrary non-verbal expression which is difficult for a physical robot. However, the most effective expression in the conversation for the virtual robot is not apparent. As the first step, therefore, we compared the virtual robot with the physical robot under the same conditions. It is noteworthy that the current result did not suggest that the advantages of having a physical body are always shown under any conditions. There is thus scope for further study to investigate the effective expressions of the virtual robot for the elderly.

The current study assumed the potential, complex confound between the engagement of the elderly and the success of the robots' answers. Therefore, we divided the participants into two groups based on how successful the robot is in responding to a speech. We then analyzed the data to investigate the effects of embodying in each group. However, there may be still confounding in the data even after such a division. As a future endeavor, the potential confounding between the engagement and the success of the robots' answers is worth investigating by an experiment with a larger number of participants, which allows us more careful consideration of how much successful (less ambiguous) conversation is established. In this future investigation, it could be interesting to examine the effects of having a physical body to the engagement of the elderly while considering how much the robots give ambiguous answers, which may reveal more (or less) importance of having a physical body relative to having successful responses.

The number of participants in this experiment was not large. Accordingly, to prevent potential variance in the data, the order and the topics in the experiment were limited to be fixed for all participants. Therefore, to allow more reliable and precise analysis, it is worth performing further experiments with more variations in topics with more subjects.

The conversation involved with the current experiment was limited to only 15 min and was conducted just once with every participant. In other words, it is still not clear whether the positive effect of having a physical body, regarding not only the engagement but also intimacy potentially established based on it, is maintained for longer use in real-world applications such as active listening robots for the elderly in a nursing home. Therefore, we need to run field experiments for conversation in a nursing home setup. For such experiments, we need to develop functions for conversation to encourage users to interact with robots in the long term, such as interactions related to the everyday routine (de Graaf et al., 2015) and remember the user's name and past conversations (Iio et al., 2020a). Moreover, in the future, it could be interesting to investigate the words and expressions preferred by the elderly.

In such field experiments and future applications in nursing homes, we must also cope with people with cognitive impairment or dementia. However, although we did not conduct our study based on any medical criteria, the participants of the current experiment seemed to be healthy. Thus, it should be worthwhile to examine the current hypotheses on the elderly with cognitive impairments, which requires us to develop further functions for robots to sustain conversations with such people (Kopp et al., 2018).

## Conclusion and Future Work

In this study, aiming to develop a robot as a conversation partner for the elderly, we investigated whether the robot should have a physical body or a virtual body. We implemented conversation systems in which two physical or virtual robots interacted with an elderly person. We conducted an experiment with 40 participants to confirm which type of robot they would interact with more and feel closer to. The results of the experiment indicated that the elderly, who is successfully responded to by robots, engaged more in the conversation with the physical robots than the virtual robots. The effect of physical robots is expected to increase as their ability to converse with people improve in future; however, this needs to be verified in long term field experiments.

## Data Availability Statement

The raw data supporting the conclusions of this article will be made available by the authors, without undue reservation.

## Ethics Statement

The studies involving human participants were reviewed and approved by the ethics committee of Osaka University. The patients/participants provided their written informed consent to participate in this study.

## Author Contributions

TN and YY prepared the materials, collected, and analyzed the data. TN wrote the first draft of the manuscript, which all authors then commented on. The final manuscript was also written by TN and approved by all the authors.

## Conflict of Interest

MC, TA, and YI were employed by the company NTT DOCOMO, INC. TN also works at NTT DOCOMO, INC. outside of the course at Osaka University but the work is entirely unrelated to this research. The remaining authors declare that the research was conducted in the absence of any commercial or financial relationships that could be construed as a potential conflict of interest.
